# Study on the Dynamic Mechanical Response of Orthotropic Materials Under Biaxial Impact Loading

**DOI:** 10.3390/ma18245634

**Published:** 2025-12-15

**Authors:** Shumeng Pang, Weijun Tao, Haifeng Ou, Jie Liu, Jiangping Chen, Liangkun Liu, Shi Huan, Zhaodong Pan, Yiquan Huang

**Affiliations:** 1 School of Environment and Civil Engineering, Dongguan University of Technology, Dongguan 523808, China; mengps0812@hotmail.com (S.P.); ouhaifeng@dgut.edu.cn (H.O.);; 2 State Key Laboratory of Explosion Science and Safety Protection, Beijing Institute of Technology, Beijing 100081, China; 3 Earthquake Engineering Research & Test Center, Guangzhou University, Guangzhou 510006, China; 4 Hebei Innovation Center for Equipment Lightweight Design and Manufacturing, School of Mechanical Engineering, Yanshan University, Qinhuangdao 066004, China; 5 School of Smart Urban Construction, Guangzhou City Polytechnic, Guangzhou 510006, China; cjp81@126.com; 6 Dongguan Key Laboratory of Disaster Prevention and Structure Rehabilitation, Dongguan 523808, China

**Keywords:** orthotropic materials, biaxial impact, dynamic mechanical response, beech wood

## Abstract

Although the dynamic response of orthotropic materials under uniaxial impact loading has been extensively studied, their behavior under multiaxial stress states, which more accurately represent real-world blast and impact scenarios, has received limited attention. To address this gap, this study employed a self-developed biaxial impact testing apparatus to systematically investigate the dynamic mechanical behavior of beech wood, a typical orthotropic material, under three biaxial loading configurations: radial-tangential, radial-longitudinal, and tangential-longitudinal. By combining theoretical derivation with experimental data, it systematically examines stress wave propagation characteristics, strain rate effects, and anisotropy evolution under different loading paths. The results reveal that beech wood exhibits significantly distinct dynamic responses along different material orientations, with a consistent strength hierarchy: longitudinal > radial > tangential. Biaxial loading notably enhances the equivalent stress–strain response and alters the deformation mechanisms and energy absorption behavior. Furthermore, lateral confinement and multiaxial stress coupling are identified as critical factors influencing the dynamic performance. This study provides the first systematic revelation of the strain rate strengthening mechanisms and wave propagation characteristics of orthotropic materials from the perspective of multiaxial dynamic loading, thereby offering theoretical and experimental foundations for developing advanced dynamic constitutive models suitable for complex impact conditions. These findings provide important guidance for the design and evaluation of lightweight impact-resistant structures in fields such as aerospace and protective engineering.

## 1. Introduction

Engineering protective structures are frequently subjected to short-duration, high-intensity dynamic loads such as impacts during service. Under such loading conditions, materials often experience complex multiaxial dynamic stress states, and their constitutive behavior differs markedly from that under uniaxial loading conditions [1,2,3]. However, existing research has predominantly focused on uniaxial impact scenarios, and the dynamic parameters obtained fall short of meeting the design requirements of practical protective structures under multiaxial stress states. With the increasing application of advanced protective materials such as fiber-reinforced composites in aerospace and other military and civilian protective structures, accurately characterizing their dynamic mechanical behavior under complex impact loading has become crucial for new material development, structural optimization, and safety assessment. In this context, for orthotropic materials whose mechanical properties are highly dependent on loading direction, systematically revealing their dynamic response and anisotropy evolution mechanisms under multiaxial impact represents a critical research priority [4,5].

The split Hopkinson pressure bar (SHPB) technique serves as a fundamental experimental method for investigating the mechanical behavior of materials at high strain rates [6,7]. However, conventional one-dimensional SHPB devices, based on one-dimensional stress wave theory, inherently assume a uniform uniaxial stress state within specimens, thus proving inadequate for accurately characterizing materials’ complex mechanical responses under multidimensional impact loading [5,8]. Early research primarily employed confining pressure applied in one-dimensional SHPB setups to study the dynamic mechanical behavior of materials under combined static-dynamic loading conditions [9,10,11,12]. Subsequently developed dedicated biaxial/triaxial apparatuses have significantly improved the accuracy of lateral deformation measurements, thereby advancing the study of material behavior under confining pressure [13,14,15].

Various technical approaches have been proposed to achieve synchronous multiaxial impact loading. Hummeltenberg et al. [16] realized biaxial loading by constructing a two-dimensional orthogonal bar system with dual drivers, though synchronization accuracy requires further improvement. The emergence of electromagnetic drive technology [17,18,19,20,21] has provided new avenues for synchronous loading of multiple stress waves. At the mechanical design level, schemes employing a single striker bar to excite multiple incident bars must address flexural and shear wave interference during wave propagation [22,23,24,25]. To this end, the wedge-shaped dual-wave bar apparatus [5] effectively suppresses shear wave interference through wave decomposition technology and a multi-layer shim structure, ensuring both synchronization and one-dimensional wave propagation characteristics, thereby significantly enhancing experimental reliability [5].

Orthotropic materials (such as natural wood, composite materials, and additively manufactured materials) exhibit outstanding energy absorption characteristics and well-balanced mechanical properties due to their distinctive microstructures, demonstrating significant potential for application in the design of lightweight impact-resistant structures [26,27,28,29]. Beech wood, as a typical example, possesses high density and a compact texture, which contribute to its remarkable flexural and compressive strength, considerable toughness, and excellent impact resistance. Through rational structural design, efficient load transfer and energy dissipation can be achieved. Nevertheless, to fully realize their potential in practical engineering applications, further systematic investigation into their mechanical behavior under multi-field coupling conditions and the corresponding influencing factors remains essential [30].

At the fundamental research level, static and quasi-static experiments have systematically elucidated the strength characteristics and deformation mechanisms of wood along different material directions. These studies have also established quantitative relationships between key parameters—such as density, moisture content, and temperature—and mechanical performance [31,32,33], thereby laying a theoretical foundation for the optimized design of engineered wood products [34,35]. In terms of dynamic performance, the energy absorption capabilities of natural woods like Scots pine have been validated through simulations and theoretical analyses, promoting the application of lightweight, high-energy-absorbing structures in crashworthiness design for automotive, marine, and other engineering fields [36,37,38]. As the strain rate increases, the dynamic mechanical behavior of these materials under high-strain-rate conditions differs significantly from that under low-strain-rate or quasi-static loading [39,40,41,42].

Orthotropic materials exhibit fundamentally distinct mechanical responses along different material orientations (e.g., radial, tangential, and longitudinal directions in wood). Under complex multiaxial impact loading, their dynamic behavior demonstrates strong direction dependence and coupling effects, rendering conventional mechanical models based on isotropic assumptions inadequate for accurately describing the underlying response mechanisms [43]. To date, research on the constitutive relationships, strain rate sensitivity, and evolution of anisotropy in such materials under multiaxial dynamic loading remains insufficient, necessitating further in-depth exploration through systematic experimental studies and theoretical modeling [5].

To address the aforementioned research gaps, this study employs a self-designed biaxial impact loading experimental system to systematically investigate the dynamic mechanical behavior of a typical orthotropic material—beech wood—under biaxial impact loading. Specifically, biaxial impact tests are conducted on beech wood under three distinct directional combinations: radial-tangential, radial-longitudinal, and tangential-longitudinal. By integrating multidimensional stress wave theory, dynamic constitutive relations suitable for biaxial stress states and expressions for equivalent stress–strain are derived and established. This facilitates a systematic analysis of the anisotropic response characteristics and strain rate sensitivity of beech wood, as well as the influence mechanisms of lateral confinement and loading path on its dynamic mechanical properties.

## 2. Theory Derivation of Specimen Under Complex Impact Loading

**Figure 1:**
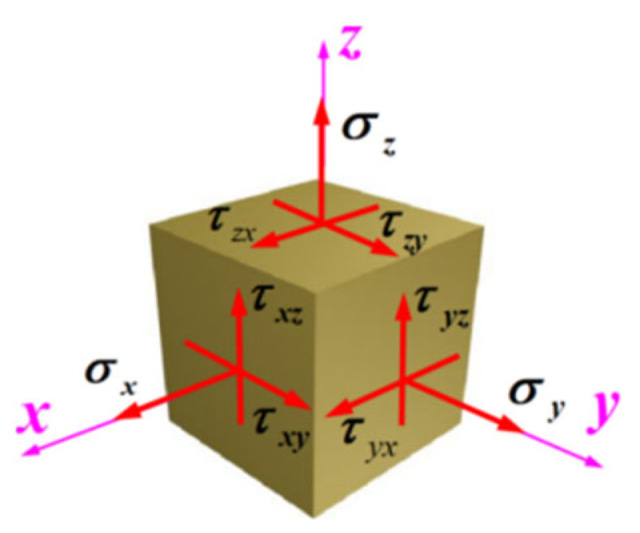
Schematic of complex forces applied to the specimen.

Engineering structures are often subjected to complex stress states, which become even more intricate under explosive or impact loading conditions, where each material unit experiences multi-axial dynamic stresses, as illustrated in Figure 1. Therefore, it is imperative to investigate the dynamic mechanical behavior and properties of engineering materials under such complex stress states.

**Figure 2:**
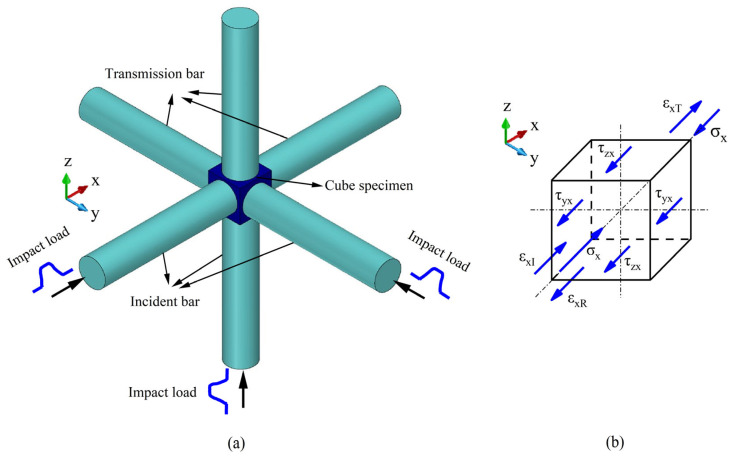
(**a**) Schematic of 3D-impact experiment, and (**b**) Schematic of forces in the axial-x direction applied on specimen.

The conventional Split Hopkinson Pressure Bar (SHPB) apparatus is designed to investigate the dynamic mechanical properties of materials under a one-dimensional stress assumption. However, when materials are subjected to multi-axial impact loading, it becomes essential to study their behavior under multi-dimensional dynamic conditions. As illustrated in Figure 2a, a cubic specimen is placed in contact with six pressure bars—forming a three-dimensional orthogonal system—where three mutually perpendicular bars serve as incident bars, and the opposite three function as transmission bars.

An identical impact stress wave is applied simultaneously to the end face of each incident bar. These three incident stress waves propagate to the bar-specimen interfaces, where reflection and transmission occur. As a result, a portion of the stress wave in each incident bar is reflected, while the remainder is transmitted across the specimen into the corresponding transmission bar. This mechanism enables three-dimensional impact loading on the specimen.

During testing, the three incident stress waves arrive at the specimen interface simultaneously. Since the loading directions of these waves are mutually perpendicular, the resultant particle motion within the specimen is determined by their combined action. The movement of the specimen along all three loading directions inevitably induces lateral motion in the bars. Consequently, the interaction between the specimen and the bars generates both longitudinal and shear stress waves.

In experiments, the propagation signals of stress waves are typically obtained by measuring voltage signals from strain gauges attached to the pressure bars. These signals conventionally correspond to longitudinal stress waves. However, during three-dimensional impact tests, the stress waves propagating in the pressure bars comprise coupled longitudinal and shear components. It is therefore necessary to separately acquire the signals corresponding to both longitudinal and shear stress waves for subsequent data analysis. Alternatively, appropriate experimental techniques must be employed to eliminate the propagation of shear stress waves, thereby facilitating more accurate interpretation of the test results.

As illustrated in Figure 2b, the forces acting on the specimen in the x-direction are analyzed. The shear stresses generated at the four side interfaces of the specimen through contact with the pressure bars are denoted as $\tau_{\mathrm{yx}}(t)$ and $\tau_{\mathrm{zx}}(t)$, respectively, while the stress induced by the impact load on the specimen is denoted as $\sigma_{x}$. Accordingly, the forces $F_{x1}(t)$ applied to the specimen by the incident and reflected stress waves can be expressed as follows:

(1) $$F_{x1}\left(t\right)\;=\;\left(\sigma_{xI}\left(t\right)\;+\;\sigma_{xR}(t)\right)A_{0}\;=\;E_{0}\left(\varepsilon_{xI}\left(t\right)\;+\;\varepsilon_{xR}(t)\right)A_{0}$$

In the above equation, $\sigma_{xI}(t)$ and $\sigma_{xR}(t)$ represent the incident wave stress and the reflected wave stress in the x-direction, respectively. $\varepsilon_{xI}(t)$ and $\varepsilon_{xR}(t)$ represent the incident wave strain and the reflected wave strain in the x-direction, respectively. $A_{0}$ and $E_{0}$ represent the cross-sectional area and the elastic modulus of the pressure bar, respectively.

The output force acting on the specimen by the transmitted stress wave can be expressed as:

(2) $$F_{x2}\left(t\right)\;=\;\sigma_{\mathrm{xT}}(t)A_{0}\;=\;E_{0}\varepsilon_{xT}(t)A_{0}$$

In the above equation, $\sigma_{xT}(t)$ and $\varepsilon_{xT}(t)$ represent the transmitted wave stress and the transmitted wave strain in the x direction, respectively.

The shear force acting on the specimen by the lateral constraint can be expressed as:

(3) $$F_{\mathrm{xLateral}}\left(t\right)\;=\;2\left(\tau_{\mathrm{yx}}\left(t\right)\;+\;\tau_{\mathrm{zx}}(t)\right)A_{s}$$

In the above equation, $A_{s}$ denotes the area of one face of the cubic specimen.

During the analysis, the force equilibrium assumption requires that:

(4) $$F_{x1}\left(t\right)\;\approx F_{x2}\left(t\right)\;+\;F_{\mathrm{xLateral}}(t)$$

Thus, the x-direction stress $\sigma_{sx}(t)$ acting on the specimen can be expressed as:

(5) $$\sigma_{sx}(t)=\frac{1}{2A_{s}}\left[E_{0}\left(\varepsilon_{\mathrm{xI}}(t)+\varepsilon_{\mathrm{xR}}(t)\right)A_{0}+E_{0}\varepsilon_{\mathrm{xT}}(t)A_{0}+2\left(\tau_{\mathrm{yx}}(t)+\tau_{\mathrm{zx}}(t)\right)A_{s}\right]\;=\frac{E_{0}}{2}\left[\left(\varepsilon_{\mathrm{xI}}(t)+\varepsilon_{\mathrm{xR}}(t)+\varepsilon_{\mathrm{xT}}(t)\right)\frac{A_{0}}{A_{s}}+\frac{2}{E_{0}}\left(\tau_{\mathrm{yx}}(t)+\tau_{\mathrm{zx}}(t)\right)\right]$$

Similarly, the stresses $\sigma_{sy}(t)$ and $\sigma_{sz}(t)$ in the y- and z-directions of the specimen can be expressed respectively as:

(6) $$\sigma_{sy}(t)=\frac{1}{2A_{s}}\left[E_{0}\left(\varepsilon_{\mathrm{yI}}(t)+\varepsilon_{\mathrm{yR}}(t)\right)A_{0}+E_{0}\varepsilon_{\mathrm{yT}}(t)A_{0}+2\left(\tau_{\mathrm{xy}}(t)+\tau_{\mathrm{zy}}(t)\right)A_{s}\right]\;=\frac{E_{0}}{2}\left[\left(\varepsilon_{\mathrm{yI}}(t)+\varepsilon_{\mathrm{yR}}(t)+\varepsilon_{\mathrm{yT}}(t)\right)\frac{A_{0}}{A_{s}}+\frac{2}{E_{0}}\left(\tau_{\mathrm{xy}}(t)+\tau_{\mathrm{zy}}(t)\right)\right]$$

(7) $$\sigma_{sz}(t)=\frac{1}{2A_{s}}\left[E_{0}\left(\varepsilon_{\mathrm{zI}}(t)+\varepsilon_{\mathrm{zR}}(t)\right)A_{0}+E_{0}\varepsilon_{\mathrm{zT}}(t)A_{0}+2\left(\tau_{\mathrm{xz}}(t)+\tau_{\mathrm{yz}}(t)\right)A_{s}\right]\;=\frac{E_{0}}{2}\left[\left(\varepsilon_{\mathrm{zI}}(t)+\varepsilon_{\mathrm{zR}}(t)+\varepsilon_{\mathrm{zT}}(t)\right)\frac{A_{0}}{A_{s}}+\frac{2}{E_{0}}\left(\tau_{\mathrm{xz}}(t)+\tau_{\mathrm{yz}}(t)\right)\right]$$

The strain $\varepsilon_{\mathrm{si}}(t)$ in the x-, y-, z-direction acting on the specimen can be expressed as:

(8) $$\varepsilon_{si}\left(t\right)=\int_{0}^{t}\dot{\varepsilon_{\mathrm{si}}}(t)dt=-\frac{2C_{0}}{l_{s}}\int_{0}^{t}\varepsilon_{\mathrm{iR}}(t)dt$$

In the above equation, i represents the x, y and z, respectively. $\dot{\varepsilon_{\mathrm{si}}}(t)$ represents the strain rate in the x-, y-, z-direction acting on the specimen. $C_{0}$ and $l_{s}$ represent the wave speed of the bars and the length of the specimen, respectively.

Based on the yield criterion, the equivalent strain and equivalent stress for the specimen under complex stress states can be determined:

(9) $$\bar{\varepsilon_{s}}(t)=\sqrt{\frac{4}{3}J_{2}^{\prime }}$$

(10) $$\bar{\sigma_{s}}(t)=\sqrt{3J_{2}}$$

where $J_{2}^{\prime }$ and $J_{2}$ correspond, respectively, to the second invariants of the deviatoric strain and stress tensors.

(11) $$J_{2}^{\prime }=\frac{1}{6}\left[{\left(\varepsilon_{sx}(t)\text{-}\varepsilon_{sy}(t)\right)}^{2}+{\left(\varepsilon_{sy}(t)-\varepsilon_{sz}(t)\right)}^{2}+{\left(\varepsilon_{sz}(t)\text{-}\varepsilon_{sx}(t)\right)}^{2}\right]$$

(12) $$J_{2}=\frac{1}{6}\left[{\left(\sigma_{sx}(t)\text{-}\sigma_{sy}(t)\right)}^{2}+{\left(\sigma_{sy}(t)\text{-}\sigma_{sz}(t)\right)}^{2}+{\left(\sigma_{sz}(t)\text{-}\sigma_{sx}(t)\right)}^{2}\right]$$

Furthermore, the equivalent strain rate of the specimen under complex stress states can be obtained by differentiating the equivalent strain formula given in Equation (9) with respect to time:

(13) $$\bar{\dot{\varepsilon_{s}}}(t)=\sqrt{\frac{2}{9}\left[{\left(\dot{\varepsilon_{sx}}(t)\text{-}\dot{\varepsilon_{sy}}(t)\right)}^{2}+{\left(\dot{\varepsilon_{sy}}(t)-\dot{\varepsilon_{sz}}(t)\right)}^{2}+{\left(\dot{\varepsilon_{sz}}(t)-\dot{\varepsilon_{sx}}(t)\right)}^{2}\right]}$$

**Figure 3:**
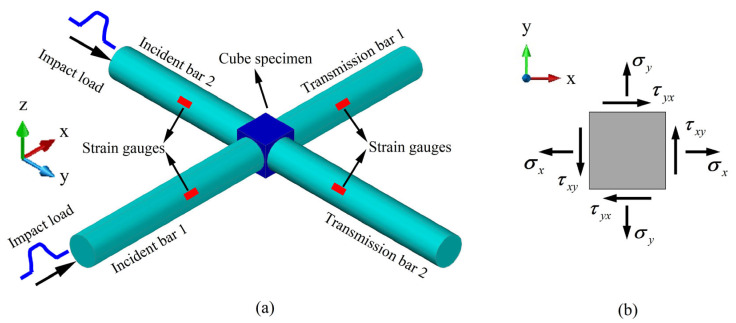
(**a**) Schematic of 2D-impact experiment [5], and (**b**) Schematic of forces applied to the specimen.

The preceding section presented the theoretical derivation for materials under three-dimensional impact loading. If the material is subjected to two-dimensional impact loading, as illustrated in Figure 3a, the cubic specimen is positioned within a two-dimensional orthogonal system consisting of two transmission bars and two incident bars. The specimen is impacted along the x- and y-directions, and a schematic of the stress state is shown in Figure 3b, where the stress in the z-direction is assumed to be zero. Based on Equations (5) and (6), the dynamic response of the material under biaxial impact loading can be derived as [5]:

(14) $$\sigma_{sx}(t)=\frac{1}{2A_{s}}\left[E_{0}\left(\varepsilon_{\mathrm{xI}}(t)+\varepsilon_{\mathrm{xR}}(t)\right)A_{0}+E_{0}\varepsilon_{\mathrm{xT}}(t)A_{0}+2\tau_{\mathrm{yx}}(t)A_{s}\right]\;=\frac{E_{0}}{2}\left[\left(\varepsilon_{\mathrm{xI}}(t)+\varepsilon_{\mathrm{xR}}(t)+\varepsilon_{\mathrm{xT}}(t)\right)\frac{A_{0}}{A_{s}}+\frac{2}{E_{0}}\tau_{\mathrm{yx}}(t)\right]$$

(15) $$\sigma_{sy}(t)=\frac{1}{2A_{s}}\left[E_{0}\left(\varepsilon_{\mathrm{yI}}(t)+\varepsilon_{\mathrm{yR}}(t)\right)A_{0}+E_{0}\varepsilon_{\mathrm{yT}}(t)A_{0}+2\tau_{\mathrm{xy}}(t)A_{s}\right]\;=\frac{E_{0}}{2}\left[\left(\varepsilon_{\mathrm{yI}}(t)+\varepsilon_{\mathrm{yR}}(t)+\varepsilon_{\mathrm{yT}}(t)\right)\frac{A_{0}}{A_{s}}+\frac{2}{E_{0}}\tau_{\mathrm{xy}}(t)\right]$$

Correspondingly, the equivalent strain and stress of the test specimen under biaxial impact loading can be derived from Equations (9) and (10) as follows:

(16) $$\bar{\varepsilon_{s}}(t)=\sqrt{\frac{4}{3}J_{2}^{\prime }}$$

(17) $$\bar{\sigma_{s}}(t)=\sqrt{3J_{2}}$$

(18) $$J_{2}^{\prime }=\frac{1}{6}\left[{\left(\varepsilon_{sx}(t)\text{-}\varepsilon_{sy}(t)\right)}^{2}+{\left(\varepsilon_{sy}(t)\right)}^{2}+{\left(\varepsilon_{sx}(t)\right)}^{2}\right]$$

(19) $$J_{2}=\frac{1}{6}\left[{\left(\sigma_{sx}(t)\text{-}\sigma_{sy}(t)\right)}^{2}+{\left(\sigma_{sy}(t)\right)}^{2}+{\left(\sigma_{sx}(t)\right)}^{2}\right]$$

Similarly, the equivalent strain rate of the specimen under biaxial impact loading can be obtained from Equation (13) as:

(20) $$\bar{\dot{\varepsilon_{s}}}(t)=\sqrt{\frac{2}{9}\left[{\left(\dot{\varepsilon_{sx}}(t)-\dot{\varepsilon_{sy}}(t)\right)}^{2}+{\left(\dot{\varepsilon_{sy}}(t)\right)}^{2}+{\left(\dot{\varepsilon_{sx}}(t)\right)}^{2}\right]}$$

## 3. Materials and Methods

### 3.1. Test Materials

**Figure 4:**
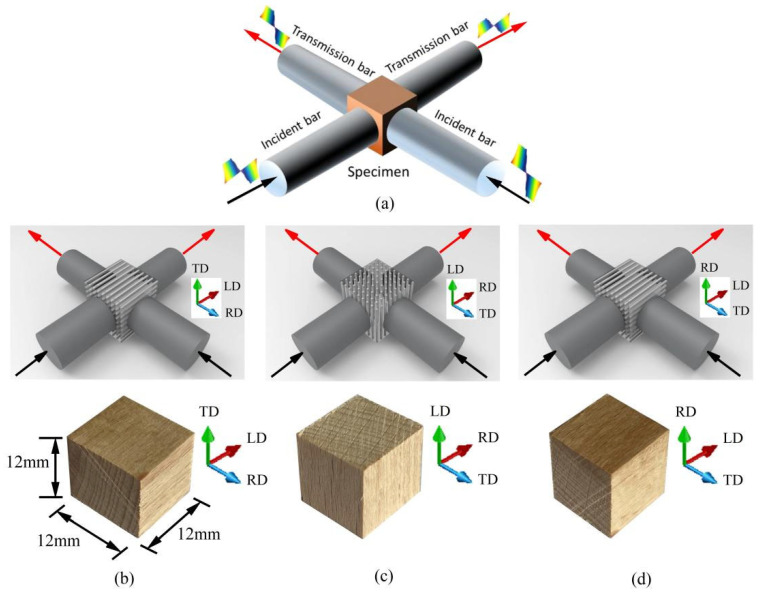
(**a**) Schematic diagram of biaxial impact test, (**b**) Beech wood specimen under radial and longitudinal loading, (**c**) Beech wood specimen under radial and tangential loading, and (**d**) Beech wood specimen under longitudinal and tangential loading [5].

This study employs naturally grown beech wood as the test material, which exhibits macroscopic orthotropic behavior with three mutually perpendicular material directions: radial direction (RD), tangential direction (TD), and longitudinal direction (LD). To ensure consistency and comparability, all specimens were extracted from the same longitudinal position within the middle section of the tree trunk and were machined along these three principal directions into cubic specimens with uniform dimensions of 12 mm × 12 mm × 12 mm, as schematically illustrated in Figure 4b–d [5].

To investigate the effects of loading direction and strain rate on the dynamic mechanical behavior of beech wood, this study was designed with three distinct loading directions (Figure 4b–d) and seven strain rate levels. For each unique testing condition—that is, each combination of loading direction and strain rate—five valid replicate specimens were used. Accordingly, a total of 35 specimens were required per loading direction (7 strain rates × 5 replicates), resulting in the preparation of 105 beech wood specimens in total for the entire experimental program. Under laboratory conditions, The measured moisture content and density of the beech wood specimens were approximately 10% and 0.73 g/cm^3^, respectively.

### 3.2. Experimental Apparatus

**Figure 5:**
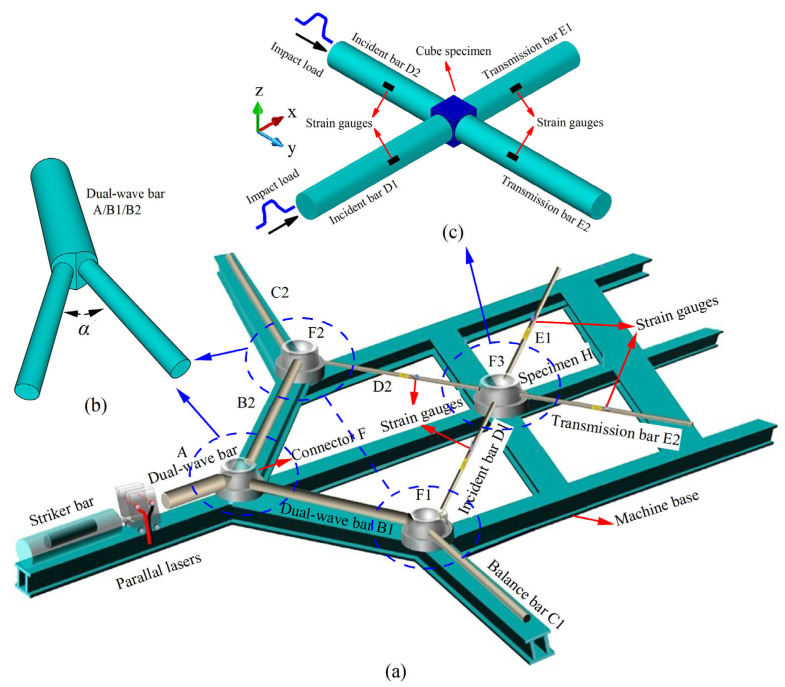
(**a**) Overall schematic diagram of biaxial impact device, (**b**) Local schematic diagram of the connection of wedge-shaped dual-wave bars, and (**c**) Schematic diagram of the placement of the cubic specimen [5].

Following the concept of a wedge-shaped dual-wave bar (DWB) and guided by stress wave propagation analysis (Figure 5), a biaxial impact device was designed [5]. The system incorporates a launch mechanism, a striker bar, two incident bars, three DWBs, two transmission bars, two balance bars, and a comprehensive measurement system. The material properties and dimensional specifications of all pressure bars used in the setup are provided in Table 1 [5].

During the entire test illustrated in Figure 5, the cubic specimen was first positioned at the intersection of four mutually perpendicular bars: incident bar D1, incident bar D2, transmission bar E1, and transmission bar E2. The end sections of these four bars were in direct contact with the four adjacent surfaces of the specimen. The launch system, utilizing gas-driven technology, propelled the striker bar to impact the initial end section of DWB A, generating a single compressive stress wave that propagated through DWB A. Upon reaching the wedge-shaped end section of DWB A, the stress wave interacted with the contacting end sections of DWB B2 and DWB B1, resulting in wave transmission and reflection phenomena. This interaction marked the first wave decomposition at connector F, producing compressive waves that propagated simultaneously into DWB B1 and DWB B2. As these compressive waves continued to propagate, a second wave decomposition occurred at connectors F1 and F2, generating two independent and mutually perpendicular incident stress waves. These waves advanced along incident bars D1 and D2, respectively, toward the specimen. Owing to the impedance mismatch at the interface between the specimen and the pressure bars, the incident waves were partially reflected and partially transmitted at bar-specimen interfaces, thereby accomplishing the impact loading on the specimen [5].

It is noted that stress wave interactions at wedge-shaped interfaces can generate both shear and longitudinal stress wave components. The presence of shear waves can interfere with strain gauge measurements, adversely affecting the accuracy and validity of the test results. To eliminate the influence of shear stress waves, multiple layers of gaskets were inserted between the pressure bars. Lubricant was uniformly applied between the gaskets and the bar end faces. This arrangement ensured that the transmitted stress waves contained only longitudinal components, thereby guaranteeing that the signals captured by the strain gauges mounted on the bars were exclusively from longitudinal stress waves [5]. This measure was critical for ensuring the accuracy of the experimental data and the validity of the tests.

The data acquisition setup consisted of strain gauges, a laser velocimeter, an oscilloscope and a high-dynamic strain indicator. Strain gauges mounted on both the incident and transmission bars captured the stress wave pulses propagating through the bars. The dynamic strain indicator communicated these signals to the oscilloscope. The stress wave signals, displayed as voltage signals on the oscilloscope, were converted into corresponding incident strain signals, reflected strain signals, and transmission strain signals. These strain signals were then substituted into the theoretical formulas presented in Section 2 “Theory Derivation of Specimen Under Complex Impact Loading” to derive the dynamic mechanical response information of the specimen under complex impact loading.

The biaxial impact loading apparatus simultaneously generates two incident stress waves to achieve two-dimensional impact loading on the test specimen. By reducing the impact load in one direction, uniaxial impact tests can be conducted. Alternatively, the setup shown in Figure 5 can be modified to enable uniaxial impact tests under varying lateral confining pressures. Specifically, bar B2 can be shortened while maintaining its contact with the wedge-shaped surface of DWB A to ensure stability and safety during impact. Meanwhile, the balance bar C2 can be removed, and two axial pressures of different magnitudes can be applied through the two pressure bars D2 and E2 to serve as lateral confinement for the specimen. This modified configuration allows for systematic investigation of the dynamic response of materials under uniaxial impact with controlled lateral pressure.

## 4. Results and Discussion

### 4.1. Experimental Validation

**Figure 6:**
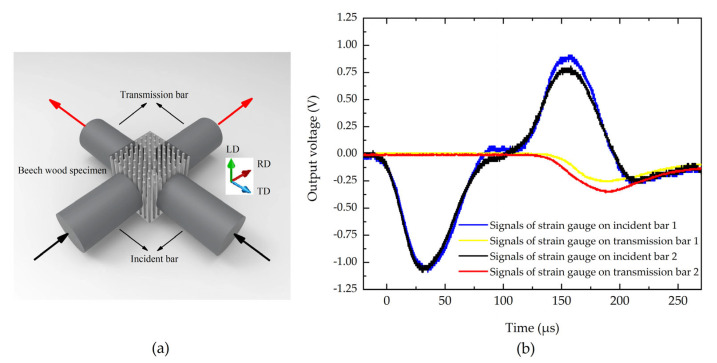
(**a**) Schematic of beech wood specimen under biaxial impact loadings, and (**b**) Acquired stress waveforms [5].

Natural beech wood was adopted as the test material, which is characterized by its macroscopic orthotropic behavior. All specimens were then processed into cubes with 12 mm sides. As illustrated in Figure 6a, the cube specimen was loaded simultaneously in the tangential and radial directions. The striker bar impacted the dual-wave bar A (DWB A) at a controlled velocity, generating an incident stress wave that propagated through the system. After two successive wave decompositions, two independent incident stress waves were produced and transmitted through the respective incident bars to load the specimen. As a result of the impedance discontinuity at the bar-specimen interfaces, the stress waves underwent both transmission and reflection. The resulting stress wave signals were captured by strain gauges mounted on both the incident and transmission bars, as depicted in Figure 6b [5].

As shown in Figure 6b, the two incident waves were initiated simultaneously and exhibited identical amplitudes, confirming the simultaneity of stress wave generation and satisfying the experimental requirements for synchronized wave loading. The transmitted waves displayed similar temporal trends but differing amplitudes, a characteristic also observed in the reflected waves. Notably, while the transmitted wave reached higher amplitudes in the radial direction, the reflected wave showed preferential amplification in the tangential direction. This directional dependence directly results from the fundamental differences in beech wood’s physical characteristics along these two principal orientations.

**Figure 7:**
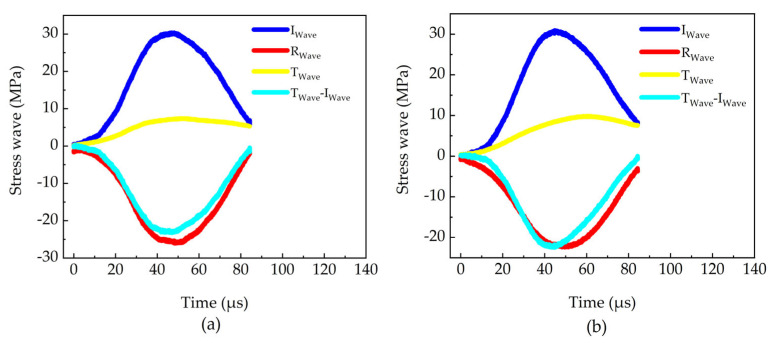
Signals of stress waves: (**a**) A three-wave equilibrium diagram in TD, and (**b**) A three-wave equilibrium diagram in RD [5].

Figure 7 further presents the waveforms of the incident, transmitted and reflected waves for both material directions. The approximate stress equilibrium achieved in each direction, as evaluated via the three-wave method, corroborates the validity and reliability of the experimental data.

**Figure 8:**
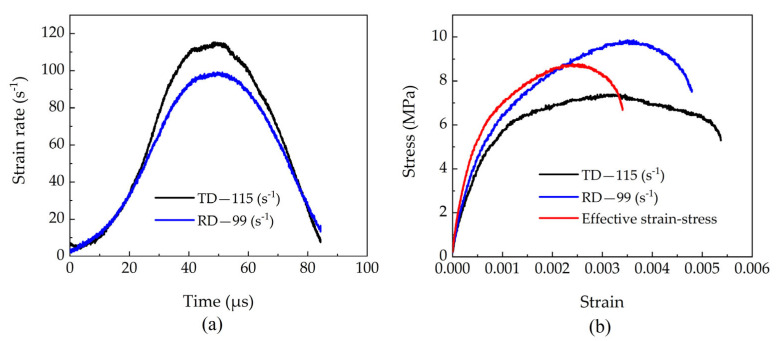
Test data results: (**a**) Strain rate-time curves, and (**b**) Strain-stress curves.

Based on the theoretical framework derived in Section 2, the strain rate–time history and stress–strain response of the specimen were calculated, as shown in Figure 8. The results indicate that the strain rate reaches approximately 115 s^−1^ under tangential loading and 99 s^−1^ under radial loading for the beech specimen.

### 4.2. Analysis and Discussion of Test Data

#### 4.2.1. Impact Loading Tests on Beech Specimens in RD and TD

As a three-dimensional orthotropic material, beech wood displays markedly distinct mechanical behaviors in the radial and tangential directions perpendicular to its fiber orientation. This section systematically examines its dynamic response under biaxial impact loading.

**Figure 9:**
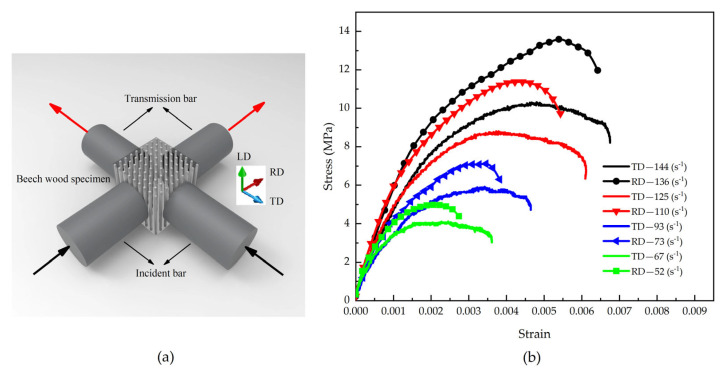
(**a**) Schematic of beech wood specimen under biaxial impact loadings, and (**b**) Stress–strain responses to biaxial impact [5].

By applying impact loading simultaneously along the tangential and radial directions of beech specimens (Figure 9a) [5], stress wave signals were captured using strain gauges mounted on the pressure bars, yielding the stress–strain curves shown in Figure 9b [5]. The results demonstrate that with increasing strain rate, both the stress amplitude and corresponding strain values increase in both the tangential and radial directions, confirming the presence of a significant strain rate effect in both material orientations. Notably, under identical testing conditions, the radial direction exhibits higher stress amplitude but lower strain values, with a correspondingly lower strain rate compared to the tangential direction.

This phenomenon can be explained by the material’s anisotropic characteristics. According to Ref. [30], beech wood exhibits higher yield strength and elastic modulus in the radial direction than in the tangential direction, indicating that the radial direction is mechanically “stiffer” than the tangential direction, with a higher wave impedance in the radial orientation. When the material is simultaneously subjected to incident stress waves of identical amplitude, the difference in wave impedance between the pressure bars and the specimen material results in less wave reflection and more wave transmission in the radial direction, while the opposite trend occurs in the tangential direction. Based on the theoretical analysis in Section 2, this wave propagation characteristic leads to a lower strain rate in the radial direction, higher overall stress amplitude, and correspondingly smaller maximum strain compared to the tangential direction.

**Figure 10:**
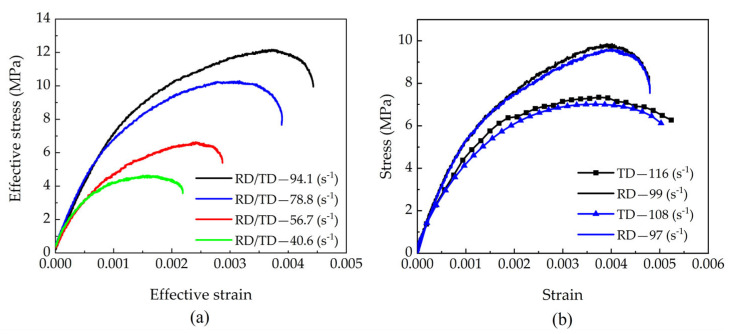
(**a**) Effective stress–strain responses to biaxial impact, and (**b**) Comparison between stress–strain responses to biaxial impact [5].

From the experimental stress–strain curves of each test group, equivalent stress–strain curves were developed. As depicted in Figure 10a [5], the equivalent ultimate strength of beech increases with the equivalent strain rate, accompanied by a corresponding rise in equivalent strain. Figure 10b compares two representative sets of tests with similar radial strain rates: while their radial stress–strain curves are essentially consistent, notable differences appear in the tangential responses. Specifically, both the tangential stress amplitude and strain values increase with rising tangential strain rates.

#### 4.2.2. Impact Loading Tests on Beech Specimens in LD and RD

**Figure 11:**
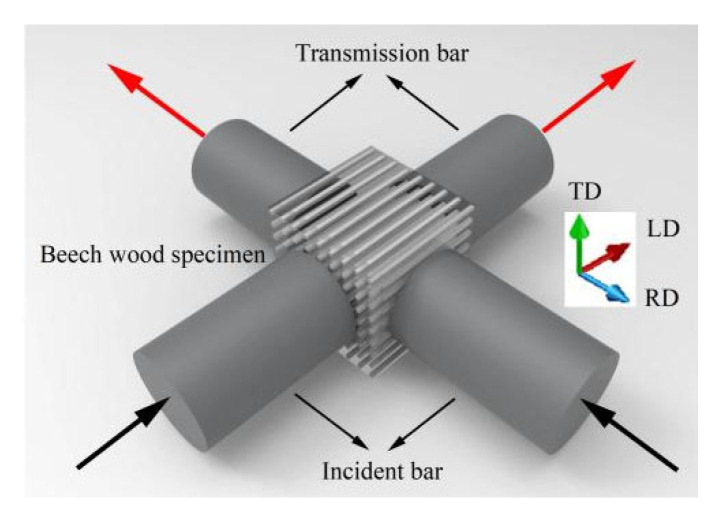
Schematic of specimen under biaxial impact loadings.

**Figure 12:**
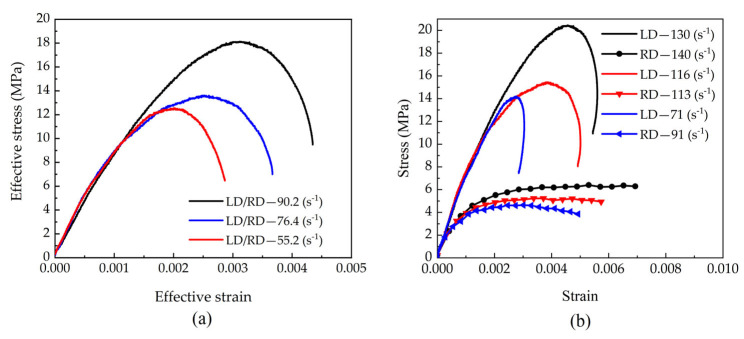
(**a**) Effective stress–strain responses to biaxial impact, and (**b**) Stress–strain responses to biaxial impact.

Figure 11 shows a schematic of the beech specimen subjected to simultaneous impact loading in the longitudinal and radial directions. Two incident loading waves were applied concurrently to the longitudinal and radial surfaces of the specimen. The test employed cubic beech specimen with a side length of 12 mm, in contact with pressure bars of 10 mm diameter. Stress wave signals were captured using strain gauges bonded to the pressure bars, and the processed data yielded a series of stress–strain curves presented in Figure 12.

From Figure 12b, it can be observed that with increasing strain rates, both the longitudinal and radial stress amplitudes show an increasing trend, accompanied by corresponding growth in strain values. It is noteworthy that within the same test group, the longitudinal stress amplitude of beech specimens consistently exceeds that in the radial direction, while the longitudinal strain is significantly smaller than the radial strain.

This phenomenon can be attributed to the material’s anisotropic characteristics. According to findings in Ref. [30], beech wood exhibits higher yield strength and elastic modulus in the longitudinal direction compared to the radial direction, with greater wave impedance in the longitudinal orientation. When the material undergoes simultaneous loading by incident stress waves of identical amplitude, differences in impedance matching conditions between the pressure bars and the material result in less wave reflection and more transmission in the longitudinal direction, while the opposite trend occurs in the radial direction. Consequently, this leads to lower strain rates in the longitudinal direction, higher overall longitudinal stress amplitudes, and relatively smaller maximum longitudinal strain values.

Based on the stress–strain curves of each test group, equivalent stress–strain curves were further derived for the specimens, as shown in Figure 12a. The results demonstrate that the equivalent ultimate strength of beech specimens significantly increases with rising equivalent strain rates, accompanied by corresponding growth in equivalent strain, collectively revealing a pronounced strain rate strengthening effect.

#### 4.2.3. Impact Loading Tests on Beech Specimens in LD and TD

**Figure 13:**
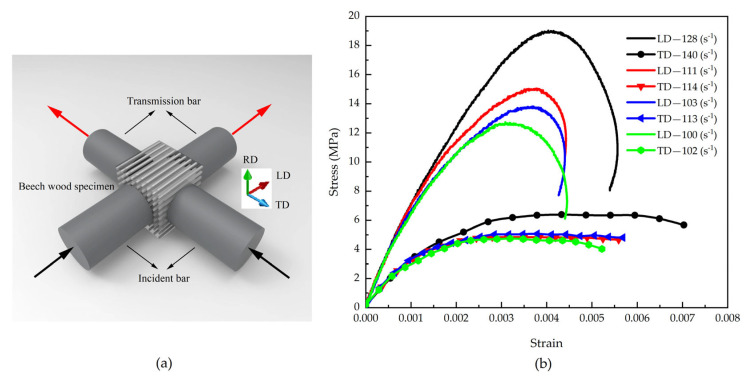
(**a**) Schematic of specimen under biaxial impact loading, and (**b**) Stress–strain responses to biaxial impact.

**Figure 14:**
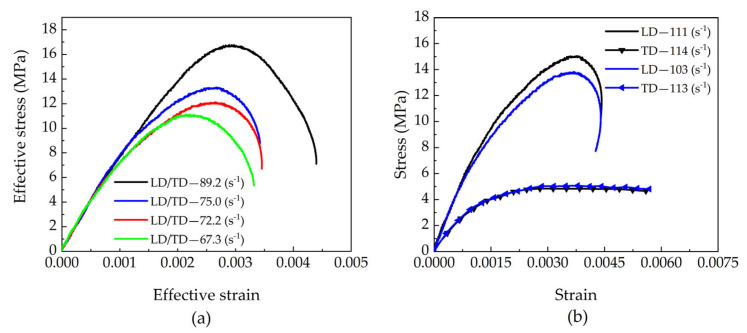
(**a**) Effective stress–strain responses to biaxial impact, and (**b**) Comparison between stress–strain responses to biaxial impact.

Figure 13a shows a schematic of the biaxial impact acting on the beech specimen in the longitudinal and tangential directions. Two incident stress waves were simultaneously applied to the longitudinal and tangential surfaces of the specimen. The test employed cubic specimens with a side length of 12 mm, in contact with pressure bars of 10 mm diameter. Recording of the stress wave signals with bar-mounted strain gauges allowed for the construction of the stress–strain curves shown in Figure 13b and Figure 14.

From Figure 13b, it can be observed that with increasing strain rates, both the longitudinal and tangential stress amplitudes show rising trends, accompanied by corresponding increases in strain amplitudes. Under identical testing conditions, the longitudinal stress amplitude of beech specimens consistently exceeds that in the tangential direction, while the longitudinal strain values are significantly smaller than the tangential strains. This phenomenon is closely related to the material’s anisotropic characteristics. According to findings in Reference [30], beech wood exhibits higher yield strength and elastic modulus in the longitudinal direction compared to the tangential direction, with greater wave impedance in the longitudinal orientation. When the material is simultaneously subjected to incident stress waves of identical amplitude, the difference in wave impedance matching conditions between the pressure bars and the material results in less wave reflection and more transmission in the longitudinal direction, while the opposite trend occurs in the tangential direction. This wave propagation characteristic leads to lower strain rates in the longitudinal direction, higher longitudinal stress amplitudes, and relatively smaller maximum longitudinal strain values.

The equivalent stress–strain curves derived from each test group are shown in Figure 14a. The results demonstrate that the equivalent ultimate strength of beech specimens significantly increases with rising equivalent strain rates, accompanied by corresponding growth in equivalent strain, collectively revealing a pronounced strain rate strengthening effect. Figure 14b further compares two test groups with similar tangential strain rates: while their tangential stress–strain curves show good agreement, notable differences appear in the longitudinal responses. Specifically, both the longitudinal stress amplitude and strain values increase with rising longitudinal strain rates, further verifying the directional dependence of the material’s mechanical properties.

**Figure 15:**
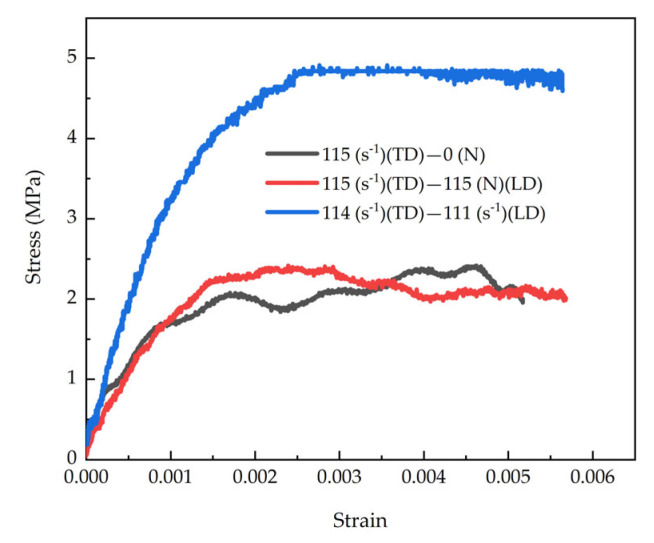
Comparison between Stress–Strain responses to different loadings.

Figure 15 presents a comparative analysis of stress–strain curves under three different loading conditions, including: uniaxial impact loading in the tangential direction, uniaxial impact loading in the tangential direction with longitudinal lateral confinement, and biaxial impact loading in both tangential and longitudinal directions. Data analysis reveals that at a constant strain rate, the stress–strain curve’s shape and evolution are governed by the lateral confinement conditions. Under biaxial impact, the tangential deformation was markedly greater than the longitudinal deformation. Moreover, the longitudinal impact further amplifies the tangential deformation, leading to a significant increase in tangential stress. Consequently, the tangential stress amplitude under biaxial impact loading is higher than that under the other two loading conditions. These results fully demonstrate that the dynamic mechanical behavior of the specimen is influenced not only by the strain rate but also significantly constrained by lateral confinement conditions and multi-axial stress states.

## 5. Conclusions

This study systematically investigated the dynamic behavior and underlying mechanisms of orthotropic materials under biaxial impact loading using a self-developed biaxial impact testing apparatus. The principal innovative findings are summarized as follows:

(1) The direction-dependent dynamic response mechanism of beech wood under biaxial impact was elucidated. Under different combined loading conditions (radial-tangential, radial-longitudinal and longitudinal-tangential), the material exhibits significantly different dynamic strength and deformation behaviors across different orientations, following the consistent strength hierarchy: longitudinal > radial > tangential. This reveals the heterogeneous and direction-coupled characteristics of orthotropic materials under complex stress states.
(2) The synergistic effect of biaxial stress states on the dynamic mechanical behavior was revealed. Experimental results demonstrate that biaxial loading not only significantly enhances the equivalent Stress–Strain response, resulting in overall curve elevation, but also alters the material’s deformation mechanisms and energy absorption modes through the coupled effects of strain rate strengthening and multiaxial stresses, thereby extending beyond the traditional mechanical understanding framework under uniaxial impact.
(3) The coupling mechanism between lateral confinement and multiaxial loading on the dynamic response was discovered. Under identical strain rate conditions, the tangential stress amplitude induced by biaxial loading is significantly higher than that under uniaxial or laterally confined uniaxial conditions, indicating that the interaction of multiaxial stress states plays a decisive role in the material’s dynamic performance. This finding demonstrates that conventional dynamic constitutive models based on isotropic assumptions are inadequate for describing the response of orthotropic materials under multiaxial impact.

This research achieves systematic innovation in experimental methodology, dynamic response characterization, and data analysis. It provides the first comprehensive revelation of the strain rate effects, wave propagation characteristics, and anisotropy evolution of orthotropic materials from the perspective of multiaxial dynamic loading. The findings establish a critical scientific basis for developing advanced constitutive models suitable for complex impact loading conditions and offer important theoretical guidance for the design and assessment of lightweight impact-resistant structures in fields such as aerospace and protective engineering.

## Figures and Tables

**Table 1:** Material parameters and bar size [5].

	Material	Density [g/cm^3^]	Elasticity Modulus [GPa]	Poisson Ratio	Diameter × Length [mm × mm]	Angle [°]
Striker bar	SUS304	7.93	193	0.3	Ø50 × 200	-
DWB A	SUS304	7.93	193	0.3	Ø40 × 1514.14	70
DWB B1/B2	SUS304	7.93	193	0.3	Ø20 × 1499.61	160
Balance bar C1/C2	SUS304	7.93	193	0.3	Ø10 × 1210	-
Incident bar D1/D2	SUS304	7.93	193	0.3	Ø10 × 1210	-
Transmission bar E1/E2	SUS304	7.93	193	0.3	Ø10 × 1210	-

## Data Availability

The original contributions presented in this study are included in the article. Further inquiries can be directed to the corresponding authors.
